# Separator‐Wetted, Acid‐ and Water‐Scavenged Electrolyte with Optimized Li‐Ion Solvation to Form Dual Efficient Electrode Electrolyte Interphases via Hexa‐Functional Additive

**DOI:** 10.1002/advs.202201297

**Published:** 2022-05-04

**Authors:** Xin Li, Jiandong Liu, Jian He, Shihan Qi, Mingguang Wu, Huaping Wang, Gaoxue Jiang, Junda Huang, Daxiong Wu, Fang Li, Jianmin Ma

**Affiliations:** ^1^ School of Physics and Electronics Hunan University Changsha 410082 China

**Keywords:** electrolyte additive, electrolytes, lithium dendrites, lithium metal batteries, solid electrolyte interphase

## Abstract

The performance of lithium metal batteries (LMBs) is determined by many factors from the bulk electrolyte to the electrode‐electrolyte interphases, which are crucially affected by electrolyte additives. Herein, the authors develop the heptafluorobutyrylimidazole (HFBMZ) as a hexa‐functional additive to inhibit the dendrite growth on the surface of lithium (Li) anode, and then improve the cycling performance and rate capabilities of Li||LiNi_0.6_Co_0.2_Mn_0.2_O_2_ (NCM622). The HFBMZ can remove the trace H_2_O and HF from the electrolyte, reducing the by‐products on the surface of solid electrolyte interphase (SEI) and inhibiting the dissolution of metal ions from NCM622. Also, the HFBMZ can enhance the wettability of the separator to promote uniform Li deposition. HFBMZ can make Li^+^ easy to be desolvated, resulting in the increase of Li^+^ flux on Li anode surface. Moreover, the HFBMZ can optimize the composition and structure of SEI. Therefore, the Li||Li symmetrical cells with 1 wt% HFBMZ‐contained electrolyte can achieve stable cycling for more than 1200 h at 0.5 mA cm^–2^. In addition, the capacity retention rate of the Li||NCM622 can reach 92% after 150 cycles at 100 mA g^–1^.

## Graphical abstract

Heptafluorobutyrylimidazole (HFBMZ) can be used as a hexa‐functional additive to remove trace H_2_O and HF from electrolyte, enhance the wettability of separator, make Li^+^ desolvated easily, and obtain stable solid electrolyte interphase (SEI) and CEI to improve the performance of lithium metal battery.

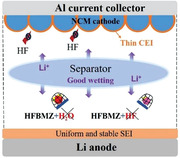

## 1 Introduction

Traditional lithium‐ion batteries (LIBs) are incapable of meeting the needs of these products for high energy density with the rapid development of portable electronic products and electric vehicles. The Li metal has lower reduction potential (−3.04 V vs standard hydrogen electrode, SHE) and higher theoretical capacity (3860 mAh g^–1^) when it was used instead of graphite anode in LIBs, making it to be an ideal anode material.^[^ ^1^, ^2^, ^3^ ^]^ However, the Li has high reactivity with electrolytes to form Li dendrites, leading to a low coulomb efficiency (CE). Besides, the Li dendrites might also puncture the separator and cause cell short‐circuits and other hazards.^[^ ^4^, ^5^, ^6^ ^]^ This problem could be aggravated by the traditional carbonate‐based electrolyte that dissolves LiPF_6_ salt. This is because the LiPF_6_ has poor hydrolytic stability, which can be decomposed easily into PF_5_ (i.e., LiPF_6_ ↔ LiF  +  PF_5_) at high temperatures. The PF_5_ is a strong Lewis acid, which can react with the H_2_O to form HF and ionic insulating POF_3_ (PF_5_  + H_2_O  →  POF_3_+ 2HF), then the generated POF_3_ can further react with H_2_O to form more ionic insulating by‐products ( POF_3_  +  H_2_O  →  POF_2_(OH)  +  HF;  POF_2_(OH)  +  H_2_O  →  POF(OH)_2_  +  HF). These by‐products have ionic insulation, which can lead to a decrease of ionic conductivity of SEI and an increase of the impedance of the cells.

In the past few decades, many methods have been developed to suppress the growth of Li dendrites, including the utilization of solid electrolytes,^[^ ^7^ ^]^ anode structure design,^[^ ^8^, ^9^ ^]^ electrolyte modification,^[^ ^10^ ^]^ and high‐concentration electrolytes,^[^ ^11^ ^]^ etc. Among them, the strategy of the electrolyte modification by additive to stabilize the Li anode is considered to be an economical and practical method. The fluorine‐containing additives (e.g., fluoroethylene carbonate (FEC)^[^ ^12^, ^13^ ^]^) can form a stable film on Li anode to inhibit dendrite growth.^[^ ^14^ ^]^ Some other additives, such as Cs^+[^ ^15^ ^]^ and K^+^ additive,^[^ ^16^ ^]^ can also suppress the Li dendrite growth by self‐repairing the electrostatic field mechanism. In addition, the Li dendrites can be also mitigated by adding additives to enhance the wettability of electrolytes and separators. For example, the electrolytes containing 0.2% polyethylene oxide‐polypropylene oxide‐polyethylene oxide (P123) can effectively enhance the wettability of the membrane, providing more Li^+^ transport channels and then promote the better distribution of Li^+^ flow on the Li anode.^[^ ^17^ ^]^ However, there is rare research on the wettability of functional electrolyte additives on the separator, let alone the combined capabilities of removing the H_2_O, capturing the HF, and mitigating the Li dendrites with optimized Li^+^ solvation and dual efficient electrode‐electrolyte interphases. Although trimethylsilyl isothiocyanate (TMSNCS)^[^ ^18^ ^]^ and 1‐(trimethylsilyl) imidazole (1‐TMSI)^[^ ^19^ ^]^ have been used to suppress the LiPF_6_ and remove the by‐products (e.g., HF and PF_5_) respectively, there is no electrolyte additive that could endow the electrolyte with aforementioned hexa‐functionalities at the same time. It is a low‐cost and more practical method by choosing an additive, which can enhance the wettability of the separator, remove H_2_O and HF, inhibit the growth of Li dendrites, optimize Li^+^ solvation, and help the formation of dual efficient electrode‐electrolyte interphases.

Herein, a completely new additive HFBMZ was developed for LMBs. The HFBMZ has the advantages of two derivatives, where the low‐polarity perfluorocarbon chain (–CF_2_CF_2_CF_3_) allows a good affinity with a non‐polar separator (polypropylene) for wetting the separator, promoting the Li^+^ pass through the separator and deposit evenly on Li metal surface. Moreover, the imidazole groups can remove the trace amounts of H_2_O and HF in the electrolyte, protecting the Li anode and inhibiting the dissolution of metal ions from the cathode NCM622 effectively. Furthermore, HFBMZ can make Li^+^ desolvated easily, which can inhibit the growth of Li dendrites effectively. In addition, HFBMZ has lower unoccupied molecular orbital (LUMO) and higher occupied molecular orbital (HOMO), can reduce and oxidize on the surface of Li anode and cathode, respectively, then a dense and stable SEI and uniform and stable CEI could be formed to enhance the cycle performance of LMBs and improve the stability of the NCM622 cathode.

## 2 Results and Discussion

**Figure 1:**
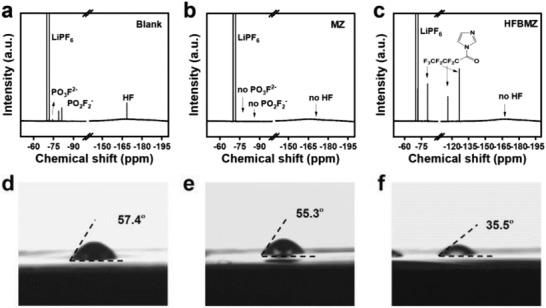
Figure 1 a–c) ^19^F NMR spectra and d–f) wettability of the separators in the blank electrolyte, MZ‐contained electrolyte, and HFBMZ‐contained electrolyte, respectively.

The capability of HFBMZ to remove H_2_O and HF from the electrolyte was demonstrated by a comparative experiment using three kinds of electrolytes (i.e., blank electrolyte, MZ‐contained electrolyte, and HFBMZ‐contained electrolyte, which contain 50 µL deionized H_2_O, respectively). Four kinds of peaks were observed in the ^19^F NMR spectrum of the blank electrolyte when the electrolyte was stored 24 h in the glovebox (**Figure** **1**a). The double peaks at 69–72 ppm correspond to the LiPF_6_, while the peaks near 75, 83, and 169 ppm are caused by the generated PO_3_F^2–^, PO_2_F^2–^, and HF,^[^ ^7^ ^]^ respectively. These results show that LiPF_6_ and PF_5_ were hydrolyzed. In contrast, there are no characteristic peaks of PO_3_F^2–^, PO_2_F^2–^ and HF observed in the ^19^F NMR when the 50 µL H_2_O was added to the 1 wt% MZ‐contained electrolyte (Figure 1b). This indicates that the MZ could absorb a small amount of H_2_O in the electrolyte and inhibit the hydrolysis of LiPF_6_. This is because the N atom in MZ has lone pair electrons, which can be easily coordinated with the H^+^ in H_2_O and HF (Figure S1, Supporting Information). The generated delocalized cations are still aromatic compounds, which can exist stably through the resonance and then achieve the goal of removing H_2_O and HF.^[^ ^19^ ^]^ Note that the characteristic peaks of PO_3_F^2–^, PO_2_F^2–^ and HF were also absent in the HFBMZ‐contained electrolyte, where only three peaks at 84, 129, and 116 ppm can be observed that belonging to the three F atoms of HFBMZ, respectively.^[^ ^20^ ^]^ These results demonstrate the capability of HFBMZ to remove H_2_O and HF. The NMR spectra of the HFBMZ‐contained electrolyte (Figure 1c) display four kinds of peaks, among which the obvious mechanism of removing H_2_O and HF may be ascribed to the C–N bond, which is unstable and easy to be broken under the condition of H_2_O and acid, releasing the MZ group. Thus, the dissociation of HFBMZ can absorb the H_2_O and HF, where the dissociated MZ could also absorb H_2_O and HF in the solution.

The wettability of the separator in different electrolytes was evaluated by the contact angles. The contact angles in the blank electrolyte, MZ‐contained electrolyte, and HFBMZ‐contained electrolyte are 57.4, 55.3, and 35.5°, respectively (Figure 1d–f). These results demonstrate that the HFBMZ‐contained electrolyte could change the wettability of the separator, while the improvement in MZ‐contained electrolyte is minor compared to that in the blank electrolyte. This should be ascribed to the low polarity of the –CF_2_CF_2_CF_3_ group, which has a good affinity with the nonpolar membrane. The –CF_2_CF_2_CF_3_ group in HFBMZ tends to contact the nonpolar membrane, then the electrolyte could spread quickly on the membrane surface^[^ ^19^ ^]^ when the HFBMZ‐contained electrolyte was dropped onto the membrane surface. The better wettability of the separator can provide more channels for Li^+^ transportation, improving the transmission efficiency of Li^+^ and also avoiding the growth of Li dendrites. Moreover, the good wettability of the separator can improve the discharge capacity, rate performance, and cycling life of the cells.^[^ ^19^, ^21^ ^]^

**Figure 2:**
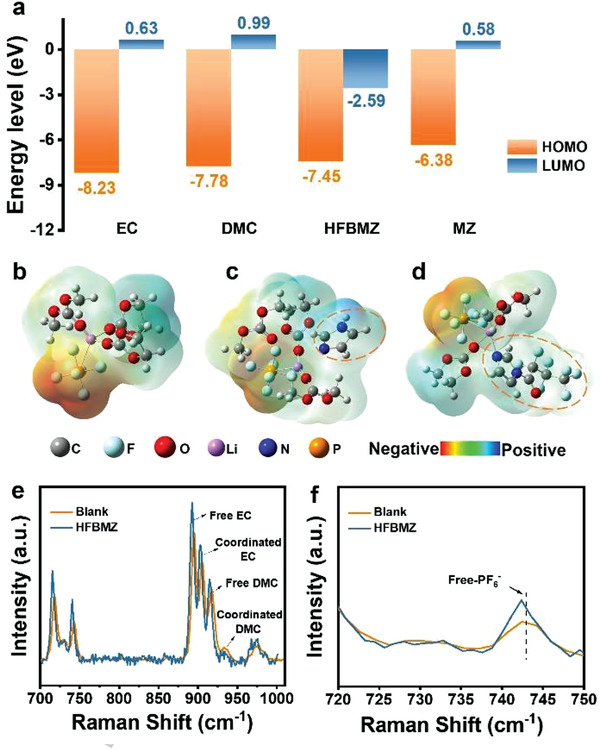
a) Theoretical calculation of HOMO (higher occupied molecular orbital) and LUMO (lower unoccupied molecular orbital) of EC, DMC, MZ, and HFBMZ molecules; The simulated primary sheath structure of Li^+^ solvation in b) LiPF_6_‐EC/DMC, c) LiPF_6_‐EC/DMC‐MZ and d) LiPF_6_‐EC/DMC‐HFBMZ electrolyte systems. Raman spectra of LiPF_6_‐EC/DMC and LiPF_6_‐EC/DMC‐HFBMZ electrolyte systems in the range of e) 700–1000 cm^–1^ and f) 720–750 cm^–1^.

The effect of additives on the Li deposition was analyzed firstly by the LUMO and HOMO calculation. It is found that the LUMO of HFBMZ (−2.59 eV) is much lower than that of EC (0.63 eV), DMC (0.99 eV), and MZ (0.58 eV) (**Figure** **2**a). This indicates that the HFBMZ could be easier to be reduced on the surface of the Li anode and participate in the formation of SEI. The addition of HFBMZ will change the interaction between Li^+^ and molecules in electrolyte, and then affect the solvation structure of Li^+^. MD simulations show that the coordination numbers of PF_6_^–^, EC, and DMC in Li^+^ solvation sheath in the blank electrolyte are 0.93, 1.93, and 1.18, respectively (Figure S2a—c, Supporting Information). The coordination number of PF^6–^ and EC in Li^+^ solvation sheath decreased by 0.09 and 0.23, while the coordination number of DMC was increased by 0.07 in MZ‐contained electrolyte. In contrast, the coordination numbers of PF_6_^–^, EC, and DMC in Li^+^ solvation sheath decreased by 0.07, 0.13, and 0.06, respectively, when HFBMZ was used as an additive (Figure 2b–d). Raman spectroscopy was used to characterize the structure of the electrolytes. As shown in Figure 2e and f, the peaks at ≈742, 894, 903, 915 and 933 cm^–1^ correspond to free PF_6_^–^, free EC, coordinated EC, free DMC, and coordinated DMC, respectively. Based on the analysis of Raman spectroscopy (Figure S2d, Supporting Information), compared with the blank electrolyte, the ratio of coordinated EC and coordinated DMC in the HFBMZ‐contained electrolyte decreases slightly, while the ratio of free EC and free DMC increases slightly. At the same time, a slight increase in free PF_6_^–^ was observed in HFBMZ‐contained electrolytes (Figure 2f). These results are consistent with those obtained by theoretical calculations. These results indicate that in the HFBMZ‐contained electrolyte, due to the decrease of the coordination number between Li^+^ and solvent anion, the binding ability between Li^+^ and solvent is weakened, resulting in Li^+^ being easier desolvated, which can increase the diffusion flux of Li^+^ on the surface of Li anode, reduce the interface resistance of Li anode, and then inhibit the formation of dendrite effectively.^[^ ^22^, ^23^ ^]^

The morphology of the deposited Li metal was characterized by SEM. It can be observed that there are various morphologies on the surface of Li anode in the blank electrolyte (Figure S3a—c, Supporting Information), in which a large amount of Li dendrites can be observed. These needle‐like Li dendrites could puncture the separator and cause internal short circuits, leading to safety problems like fire. A small amount of Li dendrites with porosity can be observed on the Li anode in MZ‐contained electrolyte, which may cause severe side reactions between electrolyte and Li, leading to continuous consumption of electrolyte. In contrast, the Li anode surface is very compact and smooth in the HFBMZ‐contained electrolyte. There are no dendrites observed. This result reveals that the HFBMZ has an obvious effect on facilitating the Li deposition and inhibiting dendrite growth.

The dynamic process of Li deposition was recorded by in situ optical microscopes. We find that the mossy Li can be observed on the surface of Li anode in the blank electrolyte within 2 min cycling (Figure S3d,e, Supporting Information), which grew longer and accumulated more with increasing the cycling time. The surface of Li metal is covered with the mossy Li after 8 min cycling, which is sufficient to pierce the separator and cause a short circuit to the cells. In contrast, there are no obvious Li dendrites observed on the surface of Li metal in the HFBMZ‐contained electrolyte even after 12 min cycling. These results further demonstrate the strong capability of HFBMZ to promote the uniform Li deposition and inhibit the Li dendrite growth, which is consistent with the observations in the SEM of Li anode in Li||Li symmetrical cells after cycling.

**Figure 3:**
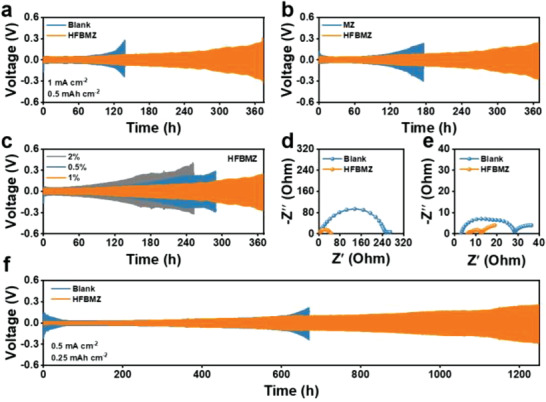
a, b) Cycling stability of Li||Li symmetric cells in the blank and HFBMZ‐contained electrolytes, and MZ‐ and HFBMZ‐contained electrolytes at 1 mA cm^–2^ with a capacity of 0.5 mAh cm^–2^; c) Cycling performance of Li||Li symmetrical cells in HFBMZ‐contained electrolytes with 0.5 wt%, 1.0 wt%, and 2.0 wt% HFBMZ, respectively; EIS spectra of the Li||Li symmetric cells with d) blank and e) HFBMZ‐contained electrolytes before and after 100 h cycles; f) Cycling performance of Li||Li symmetric cells with the blank and HFBMZ‐contained electrolytes at 0.5 mA cm^–2^ with a capacity of 0.25 mAh cm^–2^.

The cycling performance of Li||Li symmetrical cells in different electrolytes was tested to evaluate the effect of additives on Li anode. Firstly, the Li||Li symmetrical cells in the blank electrolyte started to produce voltage polarization when the cell was cycled to ~100 h, then an obvious voltage polarization appeared when the cell was cycled to ≈120 h (**Figure** **3**a). The reason for the increase in voltage polarization may be the accumulation of dead Li.^[^ ^24^ ^]^ The cycling performance of the Li||Li symmetrical cell was improved slightly (obvious polarization appeared at 150 h) when 1 wt% MZ was used as an additive (Figure 3b). In contrast, the Li||Li symmetrical cell could be stably cycled for more than 360 h when 1 wt% HFBMZ was used as an additive. This is because HFBMZ additive can produce dense and stable SEI on the surface of Li metal, which can effectively inhibit the growth of Li dendrite. The performance of Li||Li symmetrical cell using the electrolytes containing 0.5 wt%, 1 wt%, and 2 wt% HFBMZ were further tested to optimize the additive concentration (Figure 3c). It is found that the cell with 1 wt% HFBMZ electrolyte shows the most stable cycling. An uneven structure of SEI could be formed when the HFBMZ is insufficient, which cannot effectively protect Li anode. The increase of SEI impedance and polarization voltage could be observed when a high concentration of HFBMZ was used. This is because excess HFBMZ could be reduced on the surface of Li metal.

The advantages of HFBMZ‐modified electrolytes can be further verified by increasing the current density. Firstly, the Li||Li symmetrical cells can cycle for 1200 h at 0.5 mA cm^–2^ in 1 wt% HFBMZ‐contained electrolyte, which was more than twice that in the blank electrolyte (Figure 3f). Besides, the Li||Li symmetrical cells in the HFBMZ‐contained electrolyte can cycle for more than 80 h at 2 mA cm^–2^ (Figure S4a, Supporting Information), which is longer than ~50 h in the blank electrolyte. The better cycling performance both at the low and high current density confirms the positive effect of HFBMZ additives. The Li grows in‐plane at the low current density while the highly branched Li dendrites can be formed at the high current density.^[^ ^25^, ^26^ ^]^ The HFBMZ can mitigate the Li dendrite even at a high rate. The difference in the kinetic behavior of SEI formation caused by the additives can be further understood by the EIS (Figure S4c—e, Supporting Information). Two semicircles can be observed after 100 cycles in blank and HFBMZ‐contained electrolytes. The semicircle in the high‐frequency region corresponds to the impedance of Li^+^ passing through SEI (*R*_SEI_), and the charge transfer impedance (*R*_ct_) at the interface of electrode‐electrolyte in the medium frequency region.^[^ ^27^ ^]^ We find that the impedance of Li||Li symmetrical cells in the blank electrolyte before the cycle (244.8 Ω) is much higher than that in the HFBMZ‐contained electrolyte (40.3 Ω) (Figure 3d). However, the *R*_SEI_ of Li||Li symmetrical cells in the HFBMZ‐contained electrolyte (1.1 Ω) is smaller than that in the blank electrolyte (15 Ω) after 50 h cycling (Figure 3e, Figure S5a, Supporting Information). These results indicated that the stable SEI formed in HFBMZ‐contained electrolyte has a higher conductivity for transferring Li^+^.^[^ ^27^ ^]^ In addition, the *R*_ct_ of Li||Li symmetrical cells in HFBMZ‐contained electrolyte is also smaller than that in the blank electrolyte, demonstrating the fast electrochemical reaction kinetics in HFBMZ‐contained electrolytes. The test of Li||Cu half cells was carried out to further understand the polarization, cycling, and CE of the cells. In the Li||Cu half cell, the essence of CE is Li loss on the Cu collector since Li foil was used as the anode. As shown in Figure S5b, Supporting Information, in the first 60 cycles, the average CE of the cells with blank and HFBMZ‐contained electrolytes was 84% and 85%, respectively. The addition of HFBMZ slightly improved the CE. However, with the progress of the cycling, the CE of the blank electrolyte suddenly decreases, while the CE of the HFBMZ‐contained electrolyte was still stable. The same trend can be seen from Figure S5c, Supporting Information. The Li||Cu half cells with HFBMZ‐contained electrolyte show a lower polarization potential. After several electroplating/stripping cycles, the dead Li deposition in the blank electrolyte is thick, thus Li dendrites grow rapidly on the Cu surface, which increases the ion and electron diffusion resistance, and makes the polarization voltage rise rapidly. HFBMZ helps the electrolyte to form a more stable SEI, so that the cells can cycle more stably.

**Figure 4:**
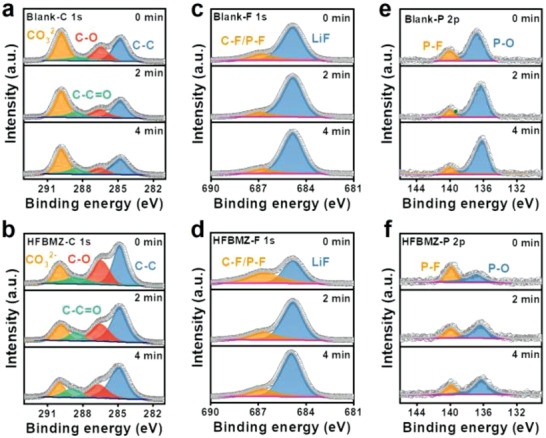
XPS spectra of Li||Li cells with the blank and 1.0 wt% HFBMZ‐contained electrolytes after 10 cycles: a, b) C 1s; c, d) F 1s; e, f) P 2p.

The effect of HFBMZ additive on the composition and structure of SEI on the surface of Li anode was further analyzed by the XPS (**Figure** **4**, Figure S6–S8, Supporting Information). Firstly, the peaks of 284.8, 286.4, 288.5, and 289.9 eV corresponding to the C–C, C–O, C–C═O, and Li_2_CO_3_ can be observed in the C 1s spectrum (Figure 4a,b), respectively. It is found that the HFBMZ additive can reduce the Li_2_CO_3_ content in SEI (Figure S6d, Supporting Information), inhibiting the EC decomposition since the Li_2_CO_3_ is the main by‐product of the EC decomposition.^[^ ^28^ ^]^ The C–F/P–F (686.7 eV) and LiF (684.8 eV)^[^ ^29^ ^]^ can be observed in spectra of F 1s (Figure 4c,d, and Figure S7, Supporting Information). With the etching time from 0 to 4 min, the content of the C–F group decreases (4.8%, 2.7%, and 2%) while the peak of LiF increases (4.95%, 8.73%, and 10.39%) in the HFBMZ‐contained electrolyte. In contrast, the changing trend of the C–F group and the peak of LiF formed in the blank electrolyte and MZ‐contained electrolyte is not obvious (Figure S8, Supporting Information). The strong polarity of the C–F bond is conducive to the adsorption of Li^+^ on the surface, while the LiF can reduce the diffusion barrier of Li^+^ on the surface and then prevent the transmission of electrons.^[^ ^23^ ^]^ Thus, this unique SEI structure is beneficial to the uniformity of Li^+^ and inhibits the formation of dendrites. In addition, the N 1s peak at 400.7 eV belonging to Li_3_N was observed in the SEI that formed in the HFBMZ electrolyte. The content of Li_3_N in SEI in HFBMZ‐contained electrolytes is 0.96%, 0.72%, and 0.73% after etching for 0, 2, and 4 min, respectively. The Li_3_N is an excellent component of SEI, which has high ionic conductivity (≈10^–3^ S cm^–1^), good electronic insulation, and can help to inhibit dendrite growth.^[^ ^30^ ^]^ Besides, the P–O (Li*_x_* POF*_y_*) peak at 136.2 eV and the P–F (Li*_x_* PF*_y_*) peak at 139.9 eV decrease obviously in HFBMZ electrolyte compared to that in the blank and the HFBMZ‐contained electrolyte (Figure 4e,f and Figure S8c,d, Supporting Information).^[^ ^31^ ^]^ This should be ascribed to the HFBMZ can remove H_2_O and HF from the electrolyte and inhibit the hydrolysis reaction of PF_5_, thus greatly reducing the P‐based by‐products in the SEI.

**Figure 5:**
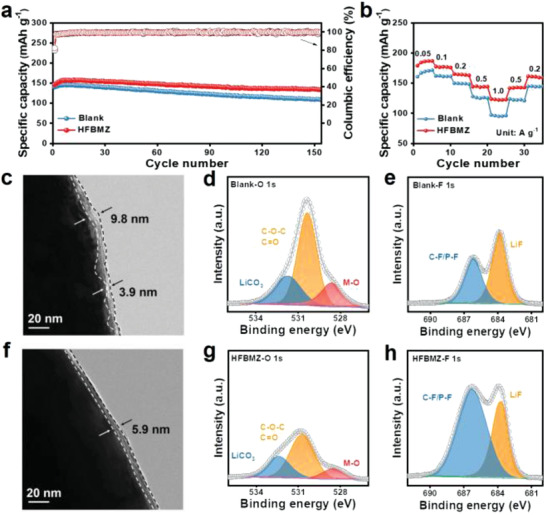
a) Cycling performance and b) rate performance of Li||NMC622 full cells in the blank and 1.0 wt% HFBMZ‐contained electrolytes; c, f) HRTEM images, d, g) XPS spectra of O 1s, and e, h) XPS spectra of F 1s of NMC622 cathode cycled in the blank and HFBMZ‐contained electrolytes.

The Li||NCM622 full cells were investigated to evaluate the practicability of HFBMZ as an electrolyte additive. Firstly, the initial capacity, the first cycle coulombic efficiency, and the capacity retention after 150 cycles are 146.2 mAh g^–1^, 82.4%, and 92% in the HFBMZ‐contained electrolytes. These values are much higher than 140 mAh g^–1^, 79.7%, and 77.8%, respectively in the blank electrolyte (**Figure** **5**a). This result can be also observed in the comparative (dis‐)charge curves in Figure S9a—b, Supporting Information. The better performance should be ascribed to the stable and unique SEI formed on the Li surface, which can inhibit the Li dendrites' growth and mitigate the electrolyte decomposition. The superiority of HFBMZ was further demonstrated by the rate performance (Figure 5b). High capacity of 180.0, 176.2, 164.2, 143.4, and 122.2 mAh g^–1^ were obtained at the current densities of 0.05, 0.1, 0.2, 0.5, and 1.0 A g^–1^ when HFBMZ electrolyte was used. These values are higher than 160.7, 161.1, 149.2, 126.1, and 95.0 mAh g^–1^ in the blank electrolyte. This result can be also observed in the comparative (dis‐)charge curves in Figure S9c,d, Supporting Information. Besides, the CV curves of Li||NCM622 full cell employing the blank electrolyte and HFBMZ‐contained electrolyte (Figure S9e—f, Supporting Information) further indicate that there is no additional oxidation peak in the HFBMZ‐contained electrolyte, verifying the stability of the electrolyte.^[^ ^32^ ^]^

The superior cycling stability of the cell can be also ascribed to the CEI formed on the NCM622, which can stabilize the structure degradation and suppress the transition metal dissolution. It was found the thickness of CEI formed on the surface of NCM622 in the blank electrolyte is uneven and loose, while the thickness of CEI on the NCM622 surface in the HFBMZ‐contained electrolyte is only 5.9 nm (Figures 5c–f). The main components of CEI were also studied by XPS to better understand the cathodic protection effect of HFBMZ (Figure S10a—b, Supporting Information). The peak of M‐O (528.7 eV) in the spectrum of O 1s indicates the dissolution of transition metals in NCM622^[^ ^33^ ^]^ (Figure 5d,g). The M‐O peak can be observed in CEI in the blank electrolyte, but it was weakened in the HFBMZ‐contained electrolyte. This indicates that the CEI formed in the HFBMZ‐contained electrolyte could inhibit the dissolution of transition metals and maintain the structural stability of NCM622. Besides, the amount of LiF in CEI increases when the HFBMZ‐contained electrolyte was employed, as shown in the F1s spectra (Figure 5e,h). This is because the HFBMZ can be easily oxidized and decomposed on the surface of the cathode due to its high HOMO, thereby resulting in an increased LiF component in CEI and a reduced diffusion barrier of Li^+^. In addition, N 1s peak in CEI was observed when the HFBMZ‐contained electrolyte was employed (Figure S10c, Supporting Information), where the Li_3_N with high ionic conductivity can further increase Li^+^ transport.^[^ ^34^ ^]^ Briefly, the HFBMZ additive can be oxidized and decomposed on the surface of NCM622 to form a thin and uniform CEI with better conductivity and faster transportability of Li^+^. In addition, the CEI film can stabilize the crystal structure of the NCM622 cathode, reduce the contact between cathode and electrolyte, inhibit the dissolution of metal ions in NCM622, and make Li‖NCM622 full cell obtain good cycling and rate performance.

## 3 Conclusions

In conclusion, we have explored HFBMZ as a new and efficient electrolyte additive to inhibit the Li dendrite growth and improve the cycling and rate performance of Li||NCM622 cells. The ability of HFBMZ to remove trace H_2_O and HF from the electrolyte and enhance the wetting capability on the separator with optimized Li ion solvation structure contributes to superior electrochemical behaviors. Moreover, HFBMZ endows LMBs with stable SEI and CEI to sustain excellent cycling performance. This work not only provides efficient hexa‐functional electrolyte additive in the community but also presents a new design thought for functional group‐directing electrolyte functions.

## Conflict of Interest

The authors declare no conflict of interest.

## Supporting information

Supporting Information

## Data Availability

The data that support the findings of this study are available in the supplementary material of this article.
