# Supplementary material for: Separator‐Wetted, Acid‐ and Water‐Scavenged Electrolyte with Optimized Li‐Ion Solvation to Form Dual Efficient Electrode Electrolyte Interphases via Hexa‐Functional Additive
Source: Adv Sci (Weinh). 2022 May 4;9(20):2201297. doi: 10.1002/advs.202201297 (PMC9284149; doi:10.1002/advs.202201297)
Supplement: Supplementary file 1 — Supporting Information [file ADVS-9-2201297-s001.pdf]

ADVANCED SCIENCE
Open Access

Supporting Information

for *Adv. Sci.*, DOI 10.1002/advs.202201297

Separator-Wetted, Acid- and Water-Scavenged Electrolyte with Optimized Li-Ion Solvation to Form Dual Efficient Electrode Electrolyte Interphases via Hexa-Functional Additive

*Xin Li, Jiandong Liu, Jian He, Shihan Qi, Mingguang Wu, Huaping Wang, Gaoxue Jiang, Junda Huang, Daxiong Wu, Fang Li and Jianmin Ma**

Supporting Information
©Wiley-VCH 2019
69451 Weinheim, Germany

Separator-Wetted, Acid- and Water- Scavenged Electrolyte Endowing Lithium Metal Battery with Efficient Electrode Electrolyte Interphases

Xin Li, Jiandong Liu, Jian He, Shihan Qi, Mingguang Wu, Huaping Wang, Gaoxue Jiang Junda Huang, Daxiong Wu, Fang Li, Jianmin Ma*

Experimental Procedures

Preparation of electrolyte and electrode

A commercial electrolyte composed of 1 M $LiPF_6$ in a mixture of ethylene carbonate (EC) and dimethyl carbonate (DMC) (1:1 in volume ratio) was used as the blank electrolyte, where the modified electrolytes were prepared by adding 0.5, 1, and 2 wt% 1-(Heptafluorobutyryl)iMidaz (HFBMZ) and 1 wt% imidazole (MZ) (>98%, Tokyo Chemical Industry Co., Ltd. (TCI Shanghai)) into the blank electrolyte, respectively. The Li foil was used as the working and counter electrode in Li||Li symmetric cells. For the full cell test, the cathode was prepared by mixing the $LiNi_{0.6}Mn_{0.2}Co_{0.2}O_2$ (NMC622) (Shanshan Co., Ltd.), poly(vinylidene fluoride) (PVDF) binder, and acetylene black (weight ratio 8:1:1) in the N-methyl pyrrolidinone (NMP) to form a uniform slurry. Then, the slurry was cast on the smooth Al foil and dried in a vacuum oven overnight. The active material loading was about 5 mg cm^{-2}. All the above electrolyte preparation and assembly of 2025 coin cells were carried out in the glove box (H_2O < 0.1 ppm, O_2 < 0.1 ppm).

Characterization and electrochemical measurements

The morphology variation of the Li foil during the Li plating/stripping process was characterized by scanning electron microscopy (SEM, Hitachi S4800). The growth of Li dendrite in Li||Li symmetric cell was observed by in-situ optical microscopy (Zoom 650 optical microscopy, Shanghai Tuming Optical Instrument Co., Ltd.). The elemental valence and compositions of the SEI layer and CEI layer were analyzed by X-ray photoelectron spectroscopy (XPS, MDTC-EQ-M20-01). The morphologies of the cathode were observed by a Transmission electron microscope (TEM, JEOLJEM-3010). 19F NMR spectrum was carried out by a nuclear magnetic resonance spectrometer (NMR, Bruker 376.5 MHz). The electrochemical test of Li||Li symmetric cells and Li||NMC622 full cells was performed by using the Neware cells testing system (CT-4008). In addition, the cyclic voltammetry (CV) and electrochemical impedance spectra (EIS) were recorded by the IVIUM electrochemical workstation.

For the electrolyte uptake experiment, the procedure was as our previous reported literature[1]. The electrolyte uptake was calculated using the following equation: Electrolyte uptake= (M – M0)/M0 ×100%, where M0 and M represent the weights of the separator before and after adsorbing electrolyte. The degree of electrolyte filling was calculated by the following equation: Degree of electrolyre filling= Ve/Vp ×100%, Ve means the volume of electrolyte absorbed by seperator. Vp represents the volume of the pores in seperator, which was confirmed by the company product.

Theoretical simulations

All the molecules were optimized by Gaussian 16 software with B3LYP/6–311+G** method and D3 dispersion correction.The classical molecular dynamics (MD) simulation of 1.0 M $LiPF_6$ in EC/DMC additive-contained electrolyte (HFBMZ, MZ) was carried out in LAMMPS[2]. First, the electrolyte was randomly placed in an 80×80×80 $Å^3$ box with an 80 ^oC bath by PACKMOL[3]. The 10^{-4} convergence criterion minimizes the initial configuration. The chamber was equilibrated in an isothermal isobaric system for 10 ns, and a Parrinello-Rahman barostat was used to maintain a pressure of 1 bar and a temperature of 300 K. The simulation was carried out in the canonical ensemble under the Nose-Hoover thermostat at 300 K for 10 ns, where the simulation time was kept long enough to reach the equilibrium state of the electrolyte system.

References

[1] J. D. Huang, J. D. Liu, J. He, M. G. Wu, S. H. Qi, H. P. Wang, F. Li, J. M. Ma, *Angewandte Chemie International Edition*, **2021**, *60*, 20717-20722.
[2] D. C. Prieve, W. B. Russel, *Journal of Colloid and Interface Science* **1988**, *125*, 1-13.
[3] L. Martínez, R. Andrade, E. G. Birgin, J. M. Martínez, *Journal of Computational Chemistry* **2009**, *30*, 2157-2164.

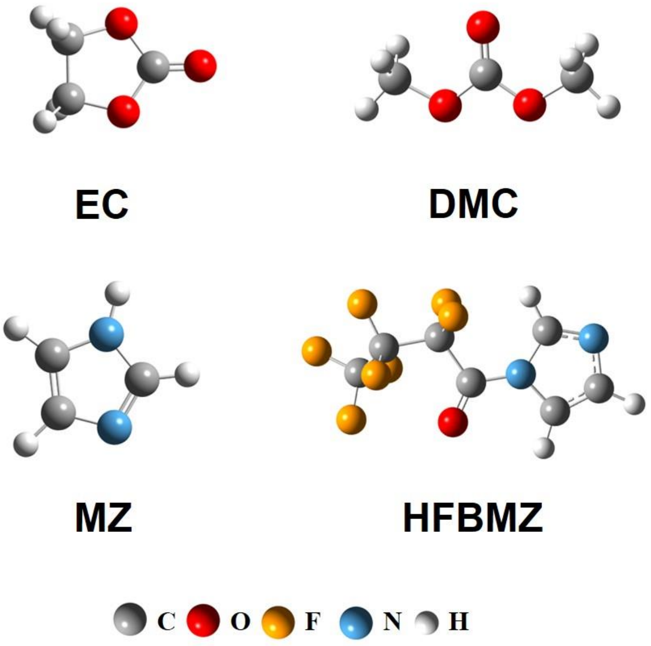

Figure S1. Chemical formula of EC, DMC, and different additives.

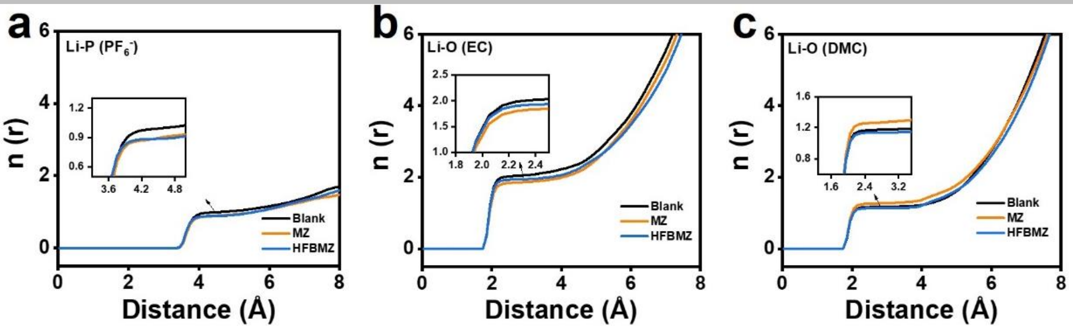

d

Raman analysis results

	Free EC	Coordinated EC	Free DMC	Coordinated DMC
Blank electrolyte	56%	44%	86%	14%
HFBMZ-contained electrolyte	58%	42%	91%	9%

Figure S2. The cumulative coordination number, donated as n (r) of Li^+ with $PF6^-$ (a), EC (b), and DMC (c). Raman analysis results of blank and HFBMZ-contained electrolyte (d).

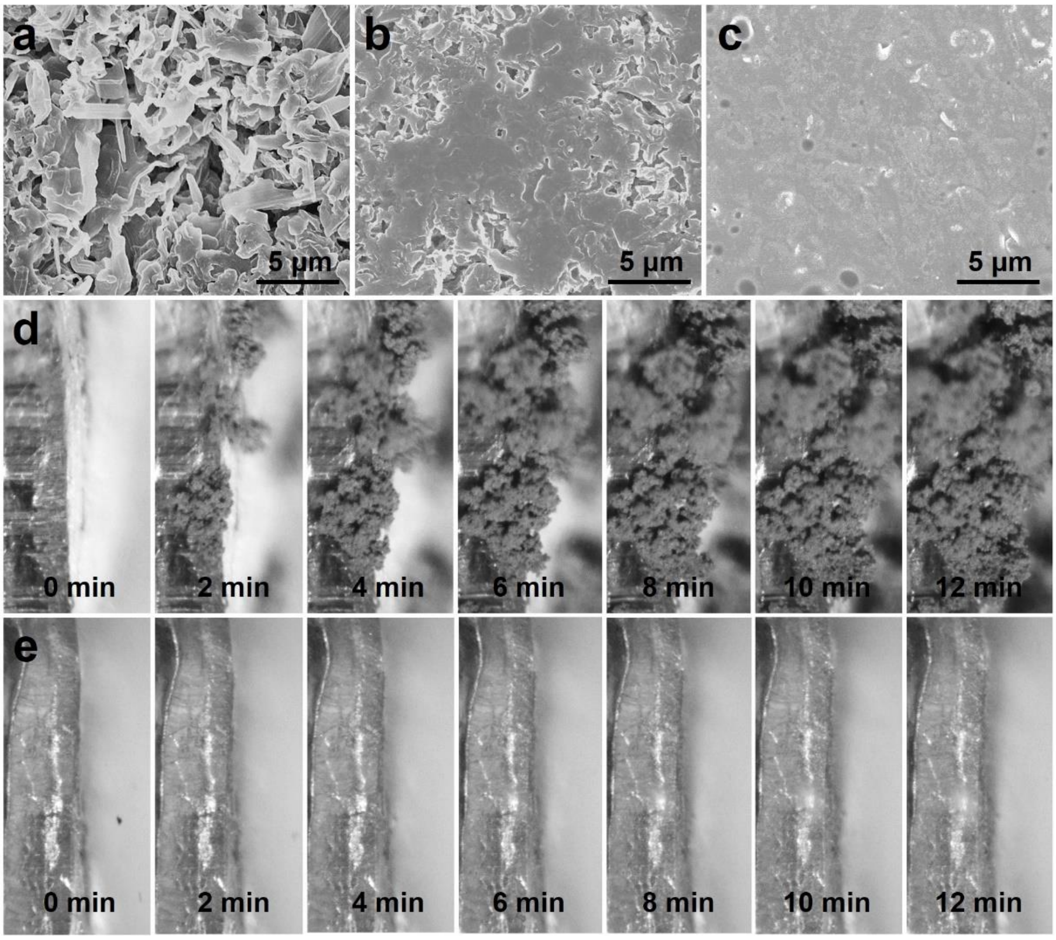

Figure S3. The SEM images of Li surface of Li||Li symmetrical cells after 50 cycles in blank electrolyte (a), MZ-contained electrolyte (b), HFBMZ-contained electrolyte (c); In situ optical microscopy was used to observe the deposition of Li in blank electrolyte (d) and HFBMZ-contained electrolyte (e).

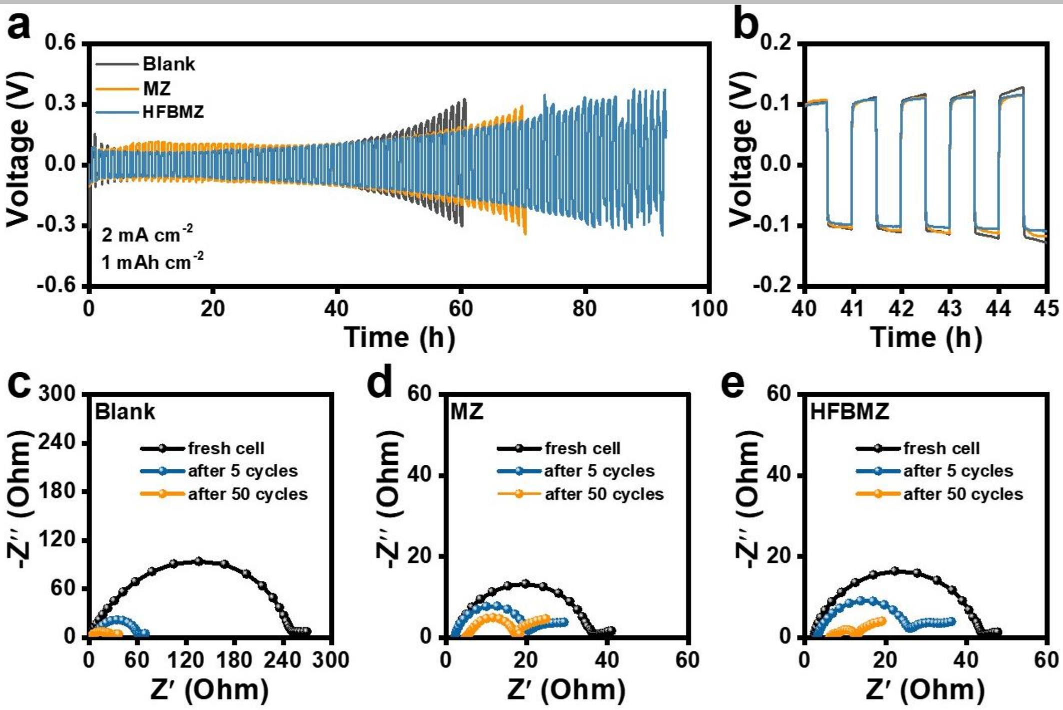

Figure S4. Cycling performance comparison (a) and detailed voltage profiles (b) of Li||Li symmetrical cells in blank electrolyte 1.0 wt% MZ-contained electrolyte, and 1.0 wt% HFBMZ-contained electrolyte at 2 mA cm^{-2} with the capacity of 1 mAh cm^{-2}. EIS graphs of the Li||Li symmetrical cells in blank (c)**,** 1.0 wt% MZ- **(**d) and HFBMZ-contained electrolyte **(e)** after 0, 5, 50 cycles at 1 mA cm^{-2} with a capacity of 0.5 mAh cm^{-2}.

a

Fitting Results			
		Blank	HFBMZ additive
Before cycle	R_b	2.1 Ω	2.6 Ω
	R_{ct}	244.8 Ω	40.3 Ω
After 50 cycles	R_b	3.8 Ω	6.5 Ω
	R_{SEI}	15.0 Ω	1.1 Ω
	R_{ct}	8.2 Ω	4.6 Ω

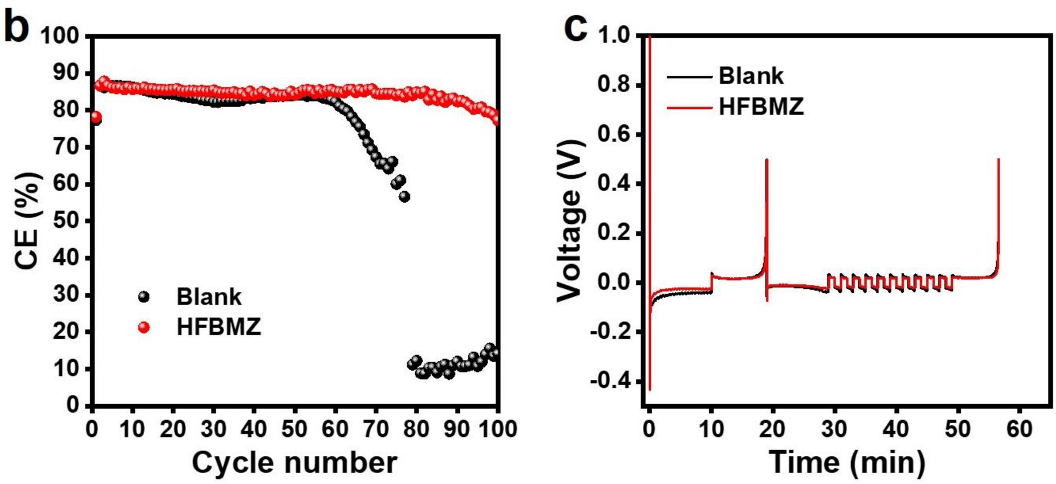

Figure S5. The equivalent circuits and the fitting results of the EIS in Figures 4d and e (a); Li plating/stripping CE in Li||Cu cells in electrolytes with/without additive (b and c).

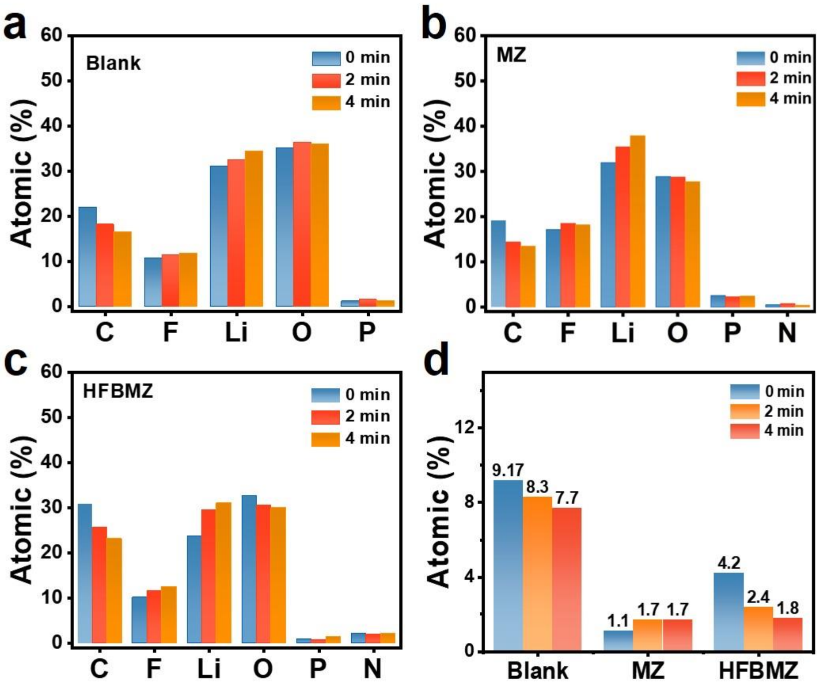

Figure S6. Atomic composition ratios of the SEI obtained by XPS spectra for electrodes cycled in blank electrolyte (a), 1.0 wt% HFBMZ- (b) and MZ-contained electrolyte (c); CO_3^{2-} group in C 1s spectra from the Li||Li cells with black, MZ-contained and HFBMZ-contained electrolyte (d).

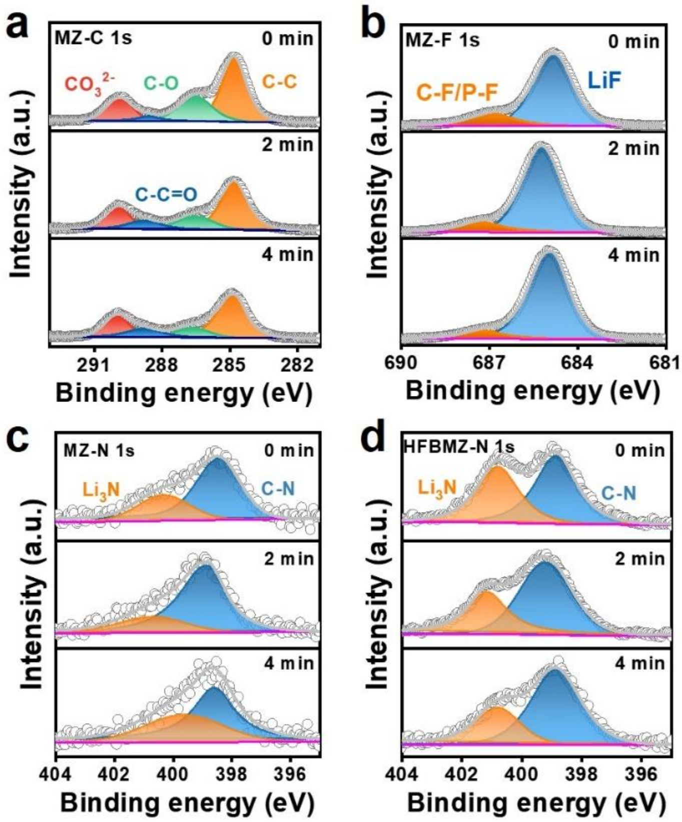

Figure S7. XPS spectra of Li||Li cells after 10 cycle test with MZ-contained electrolyte: C 1s (a); F 1s (b); N 1s (c); XPS spectra of N 1s of Li||Li cells after 10 cycle test with HFBMZ-contained electrolyte (d).

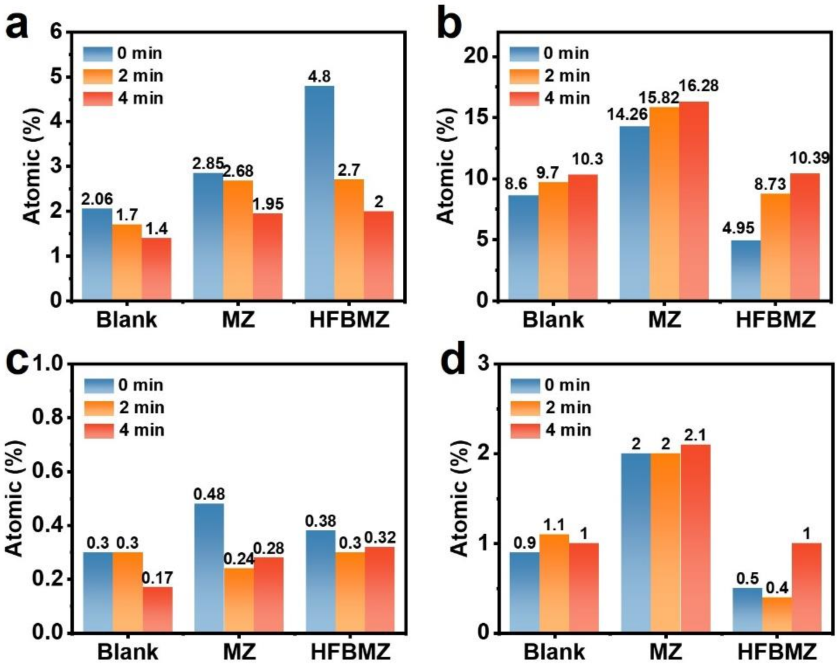

Figure S8. C-F (a) and LiF groups (b) in F 1s spectra from the Li||Li cells with black, MZ-contained, and HFBMZ-contained electrolyte; P-F (c) and P-O groups (d) in P 2p spectra from the Li||Li cells with black, MZ-contained and HFBMZ-contained electrolyte.

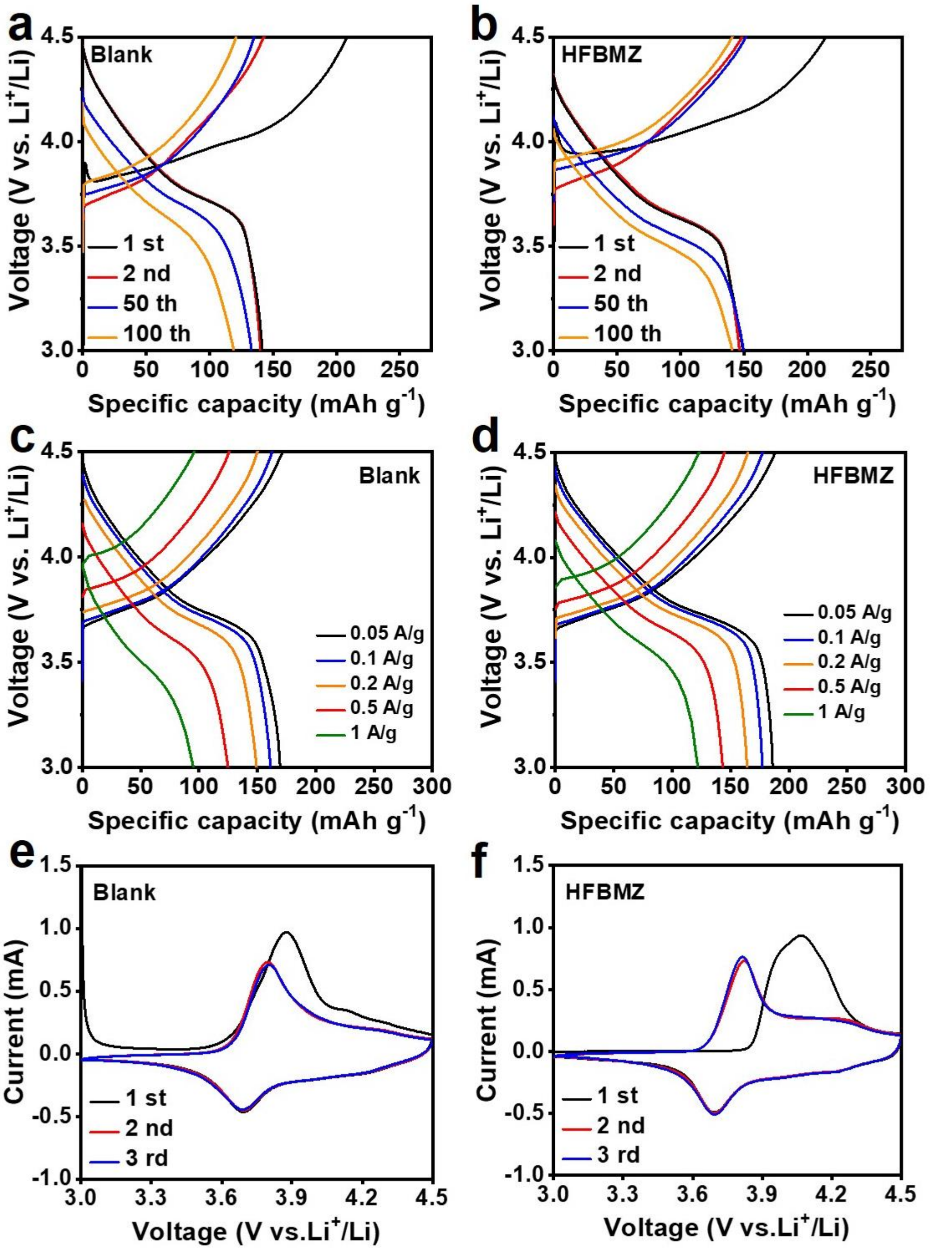

Figure S9. Charging/discharging profiles of Li||NMC622 full cells with blank electrolyte (a) and HFBMZ-contained electrolyte (b); Charging/discharging profiles under different current densities of Li||NMC622 full cells with blank electrolyte (c) and HFBMZ-contained electrolyte (d) ; CV curves of Li||NMC622 full cells with blank electrolyte (e) and HFBMZ-contained electrolyte (f) at a scan rate of 0.1 mV s^{-1}.

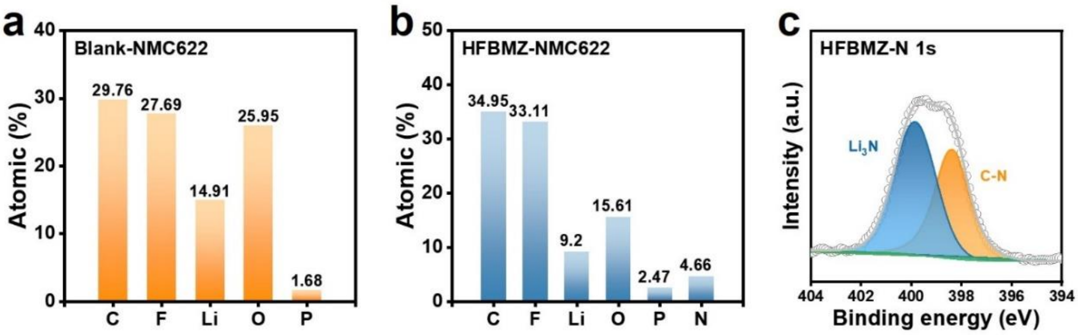

Figure S10. Atomic composition ratios of the CEI obtained by XPS spectra for electrodes cycled in blank electrolyte (a), 1.0 wt% HFBMZ- (b); XPS spectra of N 1s of Li||NMC622 full cells with HFBMZ-contained electrolyte (c).
